# Do CD4^+^ T Cell Functional Responses to Epstein–Barr Virus Provide Protective Immunity Against CNS Lymphoma in AIDS?

**DOI:** 10.1371/journal.pmed.0040110

**Published:** 2007-03-27

**Authors:** Mark A Jacobson

## Abstract

Jacobson discusses a new study addressing the question of whether an EBV antigen-specific T cell response might be a critical component of protective immunity that is lost as part of the pathogenesis of AIDS-related primary CNS lymphoma.

Since highly active antiretroviral treatment (HAART) regimens became widely available in North America and Europe in 1996, the incidence of AIDS-defining opportunistic infections and malignancies has declined dramatically. This decline has been attributed to restoration of protective immunity by HAART since HAART improves many of the deficits in T and B cell function that are caused by uncontrolled HIV infection. Some of these deficits include diminished antigen-specific T cell proliferation and cytokine expression, decreased production of new naïve T cells by the thymus, and abnormally high levels of CD4^+^ and CD8^+^ T cell and B cell activation [1–3]. However, the causal link between the reversal or prevention of these T and B cell functional abnormalities and immune protection against AIDS-defining opportunistic infections and malignancies remains largely hypothetical at present.

## Protective Immunity against CMV Retinitis in AIDS

The best evidence for HAART-induced immune reconstitution translating into protective immunity is for AIDS-related cytomegalovirus (CMV) retinitis. Before HAART, patients with CMV retinitis required lifelong anti-CMV therapy, and retinitis progressed within weeks of discontinuing therapy. However, with HAART-related increases in absolute CD4^+^ T cell counts to above 100 cells/μl, anti-CMV therapy can be discontinued with little risk of retinitis progression.

CMV antigens are well characterized, and CMV-specific immune responses are robust in healthy CMV-seropositive individuals. This means that potential correlates of protective immunity can be identified by comparing immunoassay results in patients who have clear evidence of absent CMV-protective immunity (i.e., patients with AIDS with active CMV retinitis) to those in patients who have clear evidence of restored CMV-protective immunity (i.e., patients with AIDS and CMV retinitis who, after HAART, are able to discontinue anti-CMV therapy without further retinitis reactivation or progression).

For example, we recently identified a pattern of immune function that was completely absent in the peripheral blood mononuclear cells (PBMC) of 100% of 24 patients with active CMV retinitis but was partially or completely present in the PBMC of 85% of 34 patients with CMV retinitis patients who had been clinically immuno-restored by HAART [4]. The pattern that we found was the presence of CMV antigen-specific CD4^+^ T cells that can express interleukin-2 and interferon-γ (IFN-γ) and of CMV-specific memory CD8^+^ T cells that are not fully differentiated.

Thus, specific CD4^+^ and CD8^+^ T cell functional responses to specific CMV antigens appear to be good candidates for identifying protective immunity against AIDS-related CMV disease. However, larger, longitudinal, observational studies are needed to determine the predictive value and potential clinical utility of such immunoassays in defining CMV-protective immunity and guiding clinical management for patients with AIDS with a history of CMV retinitis or at risk for developing CMV retinitis.

## The Role of EBV in Primary CNS Lymphoma

Primary central nervous system (PCNS) lymphoma is an AIDS-defining opportunistic malignancy in which Epstein-Barr virus (EBV) infection appears to have a critical pathogenic role. Like CMV retinitis, AIDS-associated PCNS lymphomas usually occur in patients with very advanced AIDS. The role of EBV in the pathogenesis of this disease is supported by studies that show the presence of EBV-encoded small RNA in tissue specimens from virtually all cases [5] and the high sensitivity and specificity of EBV DNA in cerebrospinal fluid for biopsy- or autopsy-confirmed primary CNS lymphoma [6].

However, the role of EBV viral replication and antigen-specific immune responses in the pathogenesis of primary CNS lymphoma is not as clear as that of CMV viral replication and antigen-specific immune responses in the pathogenesis of CMV retinitis. For example, CMV viral load, as measured by CMV DNA polymerase chain reaction in circulating blood or plasma, clearly correlates with risk of developing CMV retinitis; and circulating CMV DNA becomes undetectable in nearly all patients who are immune restored by HAART. In contrast, a clear relationship between circulating EBV DNA and risk of developing AIDS-related lymphoma has not been shown, and HAART has little effect in reducing circulating EBV DNA load in blood [7–9]. Analogously, nearly all studies of the effect of HAART on CMV-specific T cell responses have reported increases in these antigen-specific responses after HIV replication comes under control. However, reports of the effect of HAART on EBV-specific T cell function are mixed, with increases in certain EBV antigen-specific T cell responses and decreases in others [7,10].

## A New Study of EBV-Specific CD4^+^ T Cell Function and the Development of PCNS Lymphoma in AIDS

In a new paper published in *PLoS Medicine*, Olivier Gasser and colleagues address the question of whether an EBV antigen-specific T cell response might be a critical component of protective immunity that is lost as part of the pathogenesis of AIDS-related PCNS lymphoma [11]. The investigators performed a case-control study within the longitudinal, observational Swiss HIV Cohort Study. In six patients diagnosed with primary CNS lymphoma, EBV-specific CD4^+^ T cell IFN-γ responses were measured in PBMC obtained 0.5–4.7 y before lymphoma diagnosis, and these responses were compared to the same T cell responses in PBMC obtained from 16 matched controls (i.e., participants who were HIV positive but did not develop PCNS lymphoma).

An ELISPOT assay was used in which PBMC were stimulated by a set of EBV peptides that, based on the patients' HLA haplotype, were expected to stimulate EBV-specific memory CD4^+^ T cell IFN-γ responses, if present. In addition, CD8^+^ T cell–depleted PBMC specimens were also stimulated with EBV-infected B cell lysates in order to detect an EBV-specific CD4^+^ T cell IFN-γ response. One of the six cases versus 13 of the 16 matched controls had a detectable EBV-specific CD4^+^ T cell IFN-γ response. Of particular interest, three of the six cases developed PCNS lymphoma after being immune restored by HAART with absolute CD4^+^ T cell counts consistently above 200 cells/μl.

The strengths of this paper are the case-control method applied to a well-designed, prospective study in which samples were tested from time points before disease diagnosis. The limitations of the study are the small sample size (and thus limited generalizabililty), the absence of any EBV-specific CD8^+^ T cell functional measurements (which may be important in protective immunity), and the absence of any measurements of T or B cell activation (which are reported to have a role in the immunopathogenesis of AIDS-related non-Hodgkin's lymphoma). Also, no additional studies were done to provide insight into the mechanism by which the three immune-restored patients developed primary CNS lymphoma. Perhaps these three patients failed to reconstitute a sufficiently diverse T cell receptor repertoire to respond to critical EBV epitopes, or perhaps persistent T cell activation and apoptosis (programmed cell death) led to the loss of critical EBV-specific CD4^+^ T cell responses?

## Conclusion

Although the results of this paper are intriguing, they do not have implications for clinical practice at this time, but should stimulate new studies designed to tease out the critical components of EBV-specific protective immunity. If we can find pathogen-specific immune responses that have predictive value for defining clinical protective immunity, these responses could potentially be used in clinical practice. Such responses could become part of new clinical management strategies for determining (1) how best to clinically monitor HIV-infected patients with a history of specific opportunistic infections and malignancies or at particular risk for developing these complications, and (2) when pathogen-specific therapy for such complications should be initiated and discontinued.

## Abbreviations

CMV: cytomegalovirus
EBV: Epstein–Barr virus
HAART: highly active antiretroviral treatment
IFN-γ: interferon-γ
PBMC: peripheral blood mononuclear cells
PCNS: primary central nervous system
